# Fossorial Form of Water Voles Select and Overexploit High‐Quality Habitats, Hindering Future Colonizations Evidence From Drone‐Based Monitoring of Dandelion‐Vole Interactions in Mountain Meadows

**DOI:** 10.1002/ece3.72208

**Published:** 2025-09-22

**Authors:** Marion Buronfosse, Hélène Lisse, Geoffroy Couval, Aurélien Levret, François Gillet, Virginie Lattard, Adrien Pinot

**Affiliations:** ^1^ INRAe, VetAgro‐Sup, USC 1233 RS2GP Université de Lyon Marcy‐l'Étoile France; ^2^ Fredon Bourgogne Franche‐Comté Ecole‐Valentin France; ^3^ CNRS, UMR 6249 Chrono‐Environnement Université de Franche‐Comté Besancon France

**Keywords:** dandelion, drone, grasslands, habitat selection, remote sensing, UAV, water vole

## Abstract

Like many rodents, the water vole is able to reach high densities in meadows. During outbreaks, voles cause significant changes in plant communities. Although water voles consume a wide variety of plant species, dandelions have a unique position: they are selected by voles year‐round and serve as a key resource during winter. Voles harvest all parts of the dandelion and store the roots in almost monospecific food stores. As dandelions are perennial plants that take years to grow, vole activity can significantly affect dandelion populations. Our aim was to estimate the influence of dandelion density on vole space use, particularly habitat selection during natal dispersal. We tested the hypothesis that voles select dandelion‐rich plots for settlements. We also measured the variation in dandelion density due to new colonies' settlements to assess potential feedback effects. We hypothesized that voles decrease dandelion populations. To achieve that, we used a drone to monitor dandelions and voles over 2 years. We monitored 52 quadrats, each half a hectare, three times a year. We analyzed each image using remote sensing to locate voles and dandelions, and then examined the interactions between their locations over time. We found that dandelion‐rich plots were more likely to colonize. In plots with low dandelion density, areas denser than the plot average were also more likely to be colonized. We observed a decrease in the number of dandelions after colony settlement. Finally, we found evidence that existing burrows were more likely to be reused by new voles if dandelions were still present. This study demonstrates that dandelion density is a strong criterion in habitat selection for water voles and that vole colonies rapidly deplete this resource after establishment. These findings provide insight into plant–herbivore interactions and offer valuable perspectives for further exploration of the plant hypothesis, particularly with respect to the dynamics of resource availability and its role in cyclic population fluctuations.

## Graphical abstract

Dandelion‐rich plots are more likely to be colonized by voles. In plots with low dandelion density, areas denser than the plot average are also more likely to be colonized. We also found evidence that existing burrows are more likely to be reused by new voles if dandelions were still present.

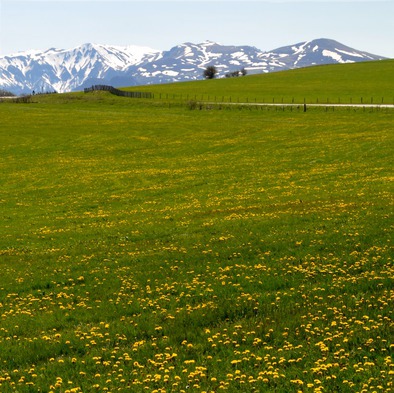

## 1 Introduction

All species have ecological requirements determined by biotic and abiotic conditions (Hutchinson 1957). To meet these requirements, animals select and utilise one or more habitats that best meet their needs. The quality of these habitats, defined as the degree to which they meet these requirements, significantly influences the survival and reproductive success of individuals, and thus the individual fitness (Franklin et al. 2000). Consequently, habitat quality selection strategies are expected to have been shaped by evolutionary processes (Morris 2003). Furthermore, non‐random spatial distribution due to habitat heterogeneity is expected. This highlights a direct link between habitat selection and the spatial distribution of individuals at the population level.

Habitat selection is a complex process involving several factors (Hutto 1985) and can be understood through theoretical frameworks. The ideal free distribution (IFD; Fretwell and Lucas 1970) theory assumes that individuals assess the food quality of all available habitat patches and preferentially occupy those with higher quality. However, a high congener density can negatively impact the trophic quality of a habitat when food is a limiting resource, leading to a balance of densities according to habitat quality. In contrast, territorial species follow the ideal despotic distribution (IDD; e.g., Fretwell 1972), where dominant individuals monopolise high‐quality resources, forcing certain individuals to select habitats of lower food quality, even while higher‐quality patches remain undersaturated. The direct and indirect effects of predation can also restrict access to certain habitats (DeCesare et al. 2014) and influence dispersal capacities (Pulliam 2000), particularly in prey species.

Rodents face several significant constraints that shape their habitat selection. As prey for many predators, their habitats must provide opportunities for effective predator avoidance (e.g., Crego et al. 2018). Additionally, rodents have low energy efficiency due to their digestive systems (e.g., Zynel and Wunder 2002) and small body size (McNab 1983), necessitating habitats with high trophic quality (e.g., Cole and Batzli 1979). However, under certain circumstances, voles are also able to colonise poor environments. They are then capable of modifying their life history traits (e.g., for *Microtus arvalis* ; Yoccoz and Ims 1999). This need is particularly crucial during winter, when the lower temperature and the higher fibre content in plants increase the energy demands. They also need to reduce or stop breeding and may change their metabolic or morphological traits (e.g., Zub et al. 2014). Furthermore, their limited dispersal capacity, compounded by the greater risk of predation with distance (Ims and Andreassen 2000), and their relatively short lifespan, impose further constraints on habitat selection. Consequently, rodents must balance these trade‐offs when selecting habitats. The ability to correctly evaluate habitat quality and select the most suitable habitat is likely favoured by natural selection.

Our study focused on whether water voles ( *Arvicola amphibius* ) assess habitat food quality when selecting territories. This species, which is widespread across various ecotypes, poses challenges for farmers in permanent mid‐altitude grasslands (above 750 m), where population outbreaks—occurring in 5‐ to 6‐year cycles (Saucy 1994)—can result in densities of more than 500–600 adults per hectare, sometimes exceeding 1500 individuals per hectare in our study area (Lisse et al. 2022). These outbreaks lead to significant disturbances in vegetation, as their burrowing activities bring soil to the surface, negatively impacting grassland yields and forage conservation. Understanding the mechanisms of habitat selection is essential to predict species distribution and population dynamics.

Water voles are ideal subjects for studying habitat selection based on food quality due to their biology and ecological behaviour. Highly territorial at the colony scale, they maintain small and stable territories throughout their lifetime (Airoldi 1976), with colonies identifiable both spatially and temporally (Airoldi and Werra 1993). Their strong antipredator adaptations, being strictly subterranean (Saucy and Schneiter 1997), allow us to focus on food‐related habitat preferences without considering antipredator habitat structure. In mid‐altitude regions, their winter survival depends heavily on their ability to store food (Potapov et al. 2004). The dandelion ( *Taraxacum officinale* ) was identified as a key food resource based on a mutlimethod study including behavioural tests (Lisse and Pinot 2024; Lisse et al. 2024). The density of dandelion varies on a scale of just a few square meters, indicating a variability in food quality within potential territories on a small scale. Given that water voles have a single dispersal event (Saucy and Schneiter 1997), settlement choices are crucial.

Our study focused on the “functional” rather than the “structural” aspects of habitat (Gaillard et al. 2010), with the aim of addressing the following questions:

Q1: Does colonisation of a new territory depend on its food quality?

Q2: Does the reuse of an old territory depend on its food quality?

Q3: Can a reduction in resources be observed after territory colonisation?

## 2 Materials and Methods

### 2.1 Study Model & Monitoring Scale

We focused on the fossorial form of the water vole ( *Arvicola amphibius* ), a species commonly found in grasslands under a continental climate. In permanent open grasslands, water voles exhibit population cycles (Saucy 1994; Giraudoux et al. 1997). During the growth phase, population density can increase from a few individuals per ha to over a hundred within 2 years (see Pascal and Boujard 1987 for a nice density curve). However, it is evident that voles are not randomly distributed during peaks, with significant variations in density observed between contiguous field plots of similar habitat.

Water voles lead an underground lifestyle, with a burrow system that is short (< 120 m) and centered around a nest (Airoldi 1976). The burrow territories are relatively stable, covering around 50 square meters and occupied by 1–3 adults (Airoldi 1976). Voles forage in the burrow by digging underground and release earth onto the surface in the form of mounds. The density of these surface mounds (referred to as surface indices) is strongly correlated with population density. This makes surface indices a reliable indicator of vole density (Giraudoux et al. 1995).

The longest distance a water vole travels on the surface is during its natal dispersal to establish its own burrow. This occurs when the water vole is between 6 and 8 weeks old and then voles stop moving on the surface (Saucy and Schneiter 1997). Field observations suggest that voles are able to occupy empty burrows, especially those of mole during their growth phase (Delattre et al. 2006).

### 2.2 Study Area & Phase of Cycle

**FIGURE 1:**
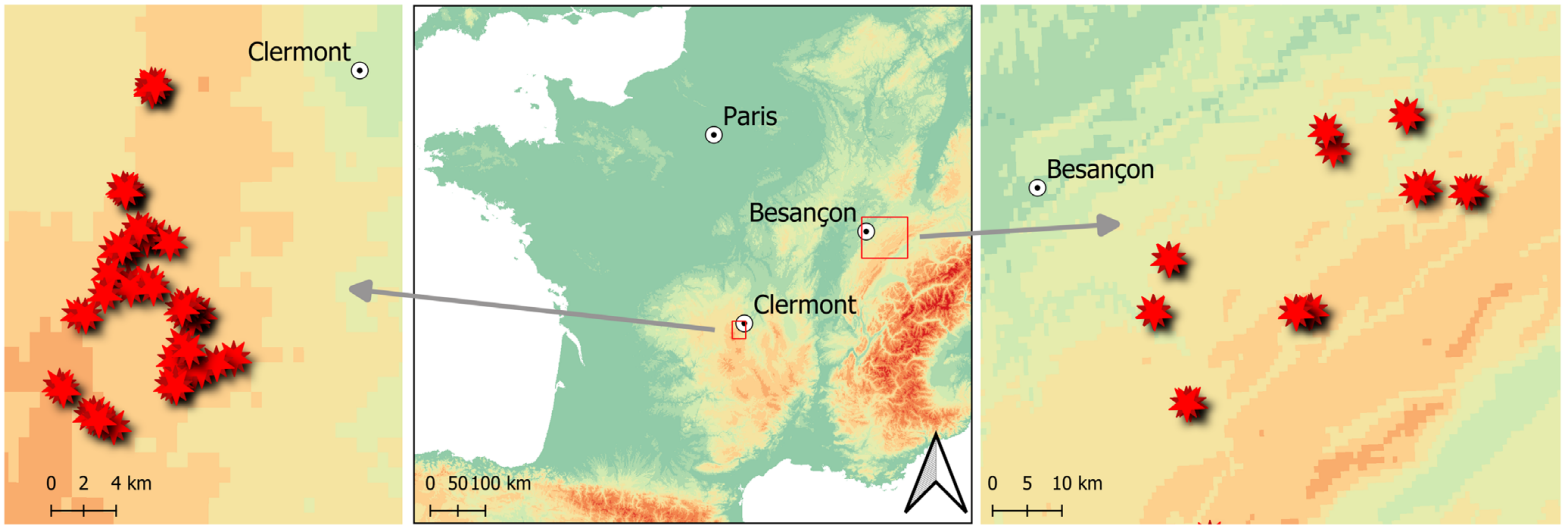
Localization of the 35 plots in Massif central (on the left) and the 19 plots in Jura Mountains (on the right). Plots are materialised with red stars.

This study focused on two regions of France with recurrent vole outbreaks since the 1970s: the Central Massif and the Jura Mountains (Figure 1). In both regions, agricultural specialisation in livestock farming has resulted in a simplified landscape mosaic favourable to vole outbreaks (Giraudoux et al. 1997). The climate is continental in both places. In the Central Massif (CM), the study site was located on the western plateau of Puy Mountain, between 850 and 1200‐m altitude. Locally, agricultural land constitutes 64% of land use that was made up of 95% of permanent grasslands in 2021 (see Appendix S1). Based on public monitoring data for voles (arvicola obs), we were able to select a subarea in the early growth phase of the cycle for positioning the study (see Appendix S1 for more methodological details). This would increase the probability that high‐quality territories would be readily available for food‐based habitat choices. According to the IDD model, we further anticipated that increasing population density (e.g., during peak periods) would reduce vole selectivity for high‐quality habitats.

In Jura Mountains, the study site was located between 400 and 900 m altitude. Locally agricultural land constitutes 56% of land use and was composed of 80% of permanent grasslands in 2021 (see Appendix S1). Dairy farming is the main farming activity in both sites with prestigious cheese production areas.

### 2.3 Sampling Design

On studied areas, 35 plots were monitored in 2020 and 2021 in MC, and 19 in Jura in 2021. The monitoring was carried out using 80 m × 60 m (4800 m^2^) quadrats, with one quadrat per field plot. The quadrats were placed more than 50 m from the nearest hedgerow and more than 20 m from the nearest field plot to avoid edge effects. The minimum distance between the adjacent quadrats was 100 m. The exact position of the quadrat within this area was chosen at random. Plots showing evidence of mole or wild boar activity were excluded to avoid interfering with the detection of surface vole indices. All field plots were mowed (spring) and grazed (fall) each year. In May 2021, the floristic composition of the CM quadrats was dominated by grasses (mainly *Poa trivialis* , *Lolium perenne* , *Dactylis glomerata* , *Trisetum flavescens* , *Holcus lanatus* ), with also a high proportion of legumes (17%, mainly *Trifolium repens* ) and *Taraxacum officinal* (13%). For each candidate field plot, rapid dandelion records were made (cover index from 0% to 5%, 5% to 10%, 10% to 25%, more than 25%). Then, plots were selected to represent a wide range of dandelion densities.

### 2.4 Monitoring Vole and Dandelion Using UAV

**FIGURE 2:**
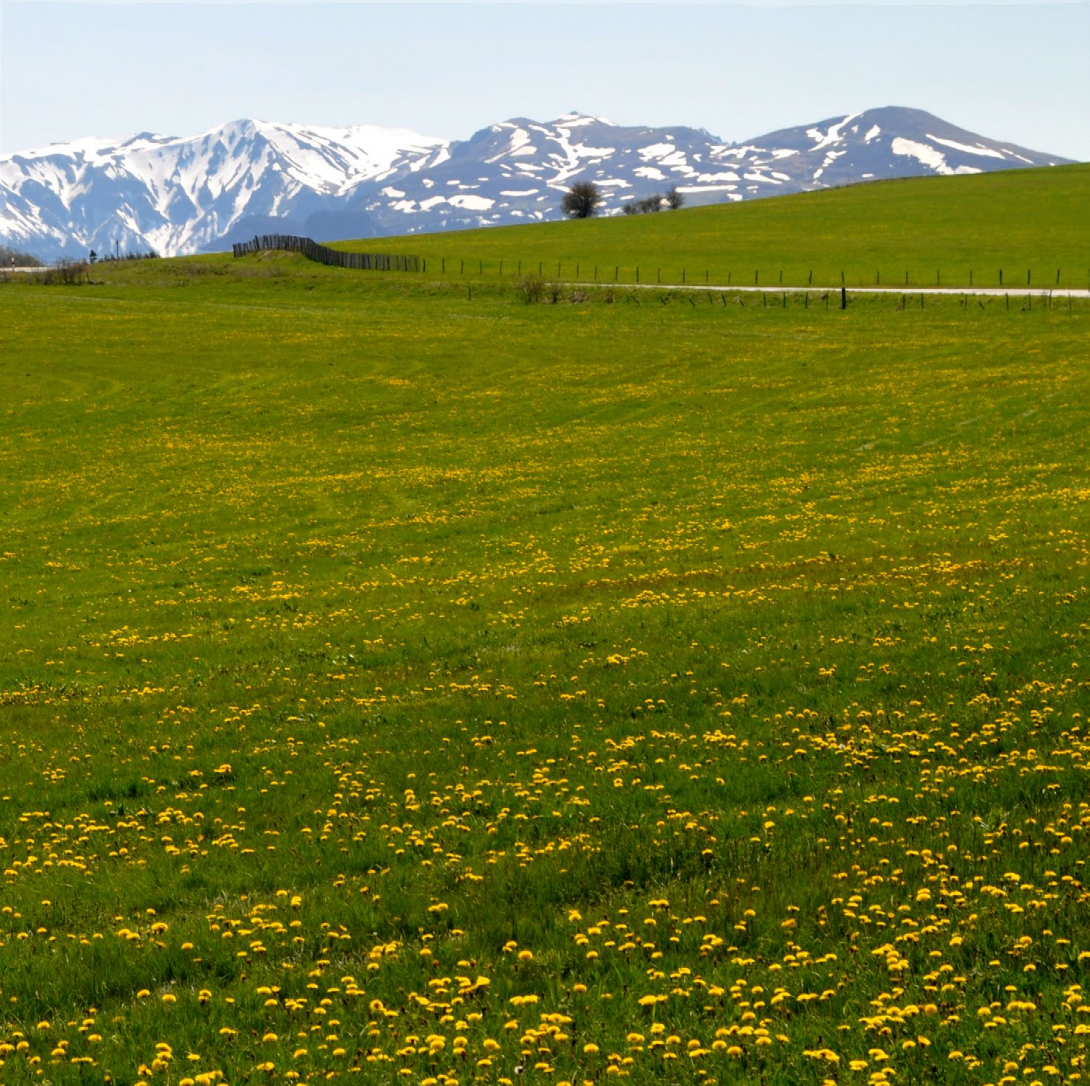
This is a picture of a monitored field plot during the flowering of dandelions.

The quadrats were monitored by drone 3 times a year to map the locations of water voles and dandelions from aerial photographs. In March, our goal was to map voles that had overwintered on the plot. In May, we aimed to map dandelions, focusing on the flowering bloom, which is synchronized by climatic conditions (Figure 2; Tanaka et al. 1988). The density of the dandelions' flowers is correlated with the biomass of the roots (see Appendix S2). In October, we aimed to map new areas colonized by voles at the end of the breeding season. For each vole picture (i.e., in March and October), the low vegetation height at these times of year allowed earth mounds to be clearly observed from the UAV. Around 50 images were taken per plot using Pix4D Capture to program the flight missions. These images were assembled into a single georeferenced picture using Pix4D Field. All the missions were carried out over a 10‐day period.

The flight altitude of the drone was set to achieve a pixel resolution of 1 cm^2^ (see Appendix S3). This resolution enables us to correctly detect dandelion flowers (~7 cm^2^ per flower head) while filtering out buttercup flowers (~2 cm^2^ per flower), which are also yellow. This also allows good detection of earth mounds. We used a sensor with three bands R: red, G: green, and B: blue (called RGB sensor).

### 2.5 Remote Sensing

To detect dandelion densities, we classified each pixel in the May image as yellow or not yellow. Yellow pixels were identified as those with an R band > 0.9, a G band > 0.9, and a B band < 0.1. To estimate vole densities, we classified each pixel as brown or non‐brown. As the color of the earth changes significantly with humidity, the RGB windows corresponding to brown earth were determined for each image.

#### 2.5.1 Broad‐Scale Index—Dataset 1

Dataset 1 summarised information into 2 m × 2 m tiles, resulting in a total of 1200 tiles per quadrat. The tile size was chosen to be much smaller than the vole burrow (e.g., the burrow system covers an area of between 20 and 60 square meters; Airoldi 1976). We recorded the cover of earth mounds and dandelions in each tile. The size of vole mounds varies (from a few square centimeters to half a square meter) depending on the location of the burrow system. The smaller ones typically have a radius of 5 cm. A tile was considered colonized by a vole if earth mounds covered more than 2.5% of the surface (equivalent to 10 mounds of 5 cm radius). The number of flower heads was estimated based on the proportion of yellow pixels only (see Appendix S3 for the image workflow). Due to the size of the pixels in comparison to the size of the flowers, buttercups (which are also yellow but much smaller) did not appear in pictures taken by the UAV. The sizes of dandelion and buttercup flowers are very consistent, with no overlap in size. The density of dandelion was evaluated by counting the number of flower heads, estimated from the area of yellow pixels, assuming that each flower head had an average surface of 7 cm^2^ (diameter 3 cm).

We compared the density of dandelion flowers in each tile with the average density of dandelion in quadrats to calculate the dandelion anomaly index. To normalize the distribution of the dandelion anomaly, a fourth root transformation was applied. Subsequently, the transformed values were centered at 0 rather than 1 to facilitate the identification of negative and positive anomalies.

#### 2.5.2 Fine‐Scale Analysis—Dataset 2

Dataset 2 was composed of 46 subplots of 20 m × 20 m centered on a colony. Colonies were identified manually and defined as groups of 15–30 earth mounds exhibiting a distinctive spatial structure and shape (easily distinguishable from those made by mole). Each colony was selected to be isolated from neighboring colonies and randomly distributed across quadrats. The 400 m^2^ area was chosen to be substantially larger than the vole territory. Of these subplots, 16 were derived from images taken in the CM in 2021, 15 in the CM in 2022, and 15 in the Jura Mountains in 2022. Using a GIS algorithm, we mapped each earth mound and each dandelion flower within these subplots (more details of remote sensing in Appendix S3).

### 2.6 Statistical Analysis

All analyzes were performed in the R environment (version 4.2.2). A forward stepwise selection procedure was used to select the model with the lowest Akaike Information Criterion (AIC). All models, except for the point pattern analysis, included the quadrat identifier as a qualitative explanatory variable (Burnham and Anderson 2002). The goodness of fit of the linear models was estimated by graphical analysis of the residuals, calculation of the explained variance, and an indicator of the collinearity thanks to the Variance Inflation Factor. The gam model was tested by the function gam.check of the MGCV package and residuals analysis.

#### 2.6.1 Dataset, Variables and Models to Answer the Q1 (Does Colonisation of a New Territory Depend on Its Food Quality?)

To assess the influence of dandelion density on site selection for new colonies, we analyzed the location of new vole colonies by comparing images taken in March and October (in 2021 and 2022 in the CM and only in 2022 in the Jura). A colony was considered as new if earth mounds were present in October but not in March. We used a subset of the dataset that included exclusively tiles that were not colonized in March (*n* = 64,537). We defined a binary response variable, where 0 indicated a non‐colonized tile and 1 indicated a colonized tile for the October picture. The anomaly of dandelion, the dandelion density in the tile, and the dandelion density in the quadrat were used as explanatory variables in a logistic regression to explain tile colonization probability.

#### 2.6.2 Dataset, Variables and Models to Answer the Q2 (Does the Reuse of an Old Territory Depend on Its Food Quality?)

We investigated the reuse of old colonies by comparing images taken in October 2021 and October 2022 (only in Massif Central). Due to agricultural practices, earth mounds are leveled at least three times a year (mowing and harrowing). A colony was considered reused if there were earth mounds in both October (voles live a couple of months) and to be abandoned if there were no more earth mounds in October the following year. We used a subset of dataset 1 that included only tiles colonized in October 2021 and March 2022 (*n* = 7851). We defined a binary response variable for colony reuse, where 0 indicated an abandoned tile and 1 indicated a tile that remained colonized in October 2022. The explanatory variables tested in the logistic regression model to explain the probability of colony reuse were local dandelion density in the tile in May 2022, average dandelion density within the quadrat in May 2022, and dandelion tile anomaly.

#### 2.6.3 Dataset, Variables and Models to Answer the Q3 (Can a Reduction in Resources Be Observed After Territory Colonization?)

##### 2.6.3.1 Broad‐Scale Pattern

To assess the effect of vole density on the variation in dandelion density in each tile, we used the logarithmic transformed dandelion population growth rate (DPGR = ln[Average number of flowers in Year 2/Average number of flowers in Year 1]) between May 2021 and May 2022 (in the CM only) as the response variable. The DPGR considers the average number of flower heads per plant to account for variations in the flowering stage between 2021 and 2022. We used a subset of dataset 1 that included only tiles that were not colonized in May 2021 (*n* = 27,852). The explanatory variable tested in linear regression was the percentage of surface of the earth mounds in March 2022 (cover of surface indices for vole colonies that have overwintered).

##### 2.6.3.2 Fine Scale Pattern

The aim of this statistical analysis was to evaluate dandelion depletion as a function of the distance from the mounds. We used point pattern analysis on dataset 2 (20 × 20 m).

The distance between the earth mounds and the dandelions was calculated using 20 consecutive buffers, ranging logarithmically from 5 cm to 5 m. The number of heads of dandelion flowers within each buffer was counted. To estimate dandelion depletion, we calculated the ratio between the number of flower heads in each buffer and the average number of flower heads in the subplot.

We used a generalized additive model (GAM, normal distribution) to predict changes in dandelion density (*DD*) as a function of the distance to the mounds (*DM*). The GAM was preferred over the linear model due to the expected nonlinear effect of distance. The colony was added as a random effect to account for the dependence among buffers.

**FIGURE 3:**
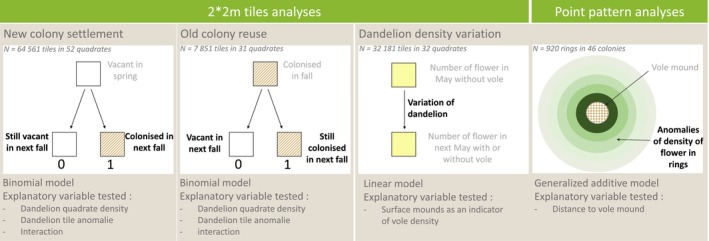
Analysis framework for vole colony dynamics and dandelion density interactions.

All hypotheses and models used in the study are summarized in Figure 3.

## 3 Results

### 3.1 Dataset Overview

Surface tumuli were measured 92 times in March over the years 2021 and 2022. In this month, the surface of the earth mounds within a quadrat was between 0.09% and 40.04%. The median was 2.01%, which represents around 615 vole tumuli of 10 cm diameter (Appendix S4: Figure S2A). Of the 35 quadrats monitored over the 2 years, 23 showed an increase in soil cover between March 2021 and March 2022, indicating an increase in vole density (Appendix S4: Figure S2B). The average dandelion cover per quadrat ranged from 0 to 58 dandelion flowers per m^2^ (Appendix S4: Figure S2C). Overall, the abundance of dandelion was higher in 2021 compared to 2022, with a median dandelion growth rate of 0.321 between these 2 years.

### 3.2 Q1: Does Colonisation of a New Territory Depend on Its Food Quality?

There were 64,537 uncolonized tiles in March in the dataset. In October, 11,734 (18.2%) of these tiles had been colonized, while the remaining 52,803 remained uncolonized. The optimal model identified the density of dandelion, the anomaly of dandelion, and its interaction as significant factors influencing site selection by voles (AIC comparison, Table 1A).

**TABLE 1:** Model selection table for the three hypotheses.

GLM equation	df	AIC	∆AIC	Explained variance
(A) Vole colonisation				
Col ~ 1 + *Q*	52	42,060	1248	0.314
Col ~ Anom + *Q*	53	41,074	262	0.330
Col ~ *D*_t_ + *Q*	53	41,812	1000	0.318
Col ~ *D*_q_ + *Q*	53	41,810	998	0.318
Col ~ *D*_t_ + *D*_t_:*D*_q_ + *Q*	54	41,788	976	0.318
Col ~ Anom + *D*_t_ + *Q*	54	41,075	263	0.331
Col ~ Anom + *D*_q_ + *Q*	54	40,868	56	0.334
Col ~ Anom*D_t_ + *Q*	55	40,959	147	0.333
Col ~ Anom*D_q_ + *Q*	55	40,812	0	0.333
(B) Colony reuse				
Reuse ~ 1 + *Q*	31	5887.8	56.2	0.429
Reuse ~ Anom + *Q*	32	5887.5	55.9	0.429
Reuse ~ *D*_t_ + *Q*	32	5854.8	23.2	0.433
Reuse ~ *D*_t_ + Anom + *Q*	33	5856.3	24.7	0.433
Reuse ~ *D*_t_*Anom + *Q*	34	5858.2	26.6	0.433
Reuse ~ *D*_t_ + *D*_t_:*D*_q_ + *Q*	33	5831.6	0	0.435
(C) Effect of vole on dandelion population growth rate (broad‐scale analysis)				
log(DGR) ~ 1 + *Q*	33	94,138	611	0.655
log(DGR) ~ Cover + *Q*	34	93,663	136	0.661
log(DGR) ~ log(Cover + 1) + *Q*	34	93,527	0	0.663
(D) Dandelion depletion by vole (fine scale pattern)				
DD ~ Subplot	47.0	−41.3	558	0.477
DD ~ S(DM)	6.5	282.68	882	0.224
DD ~ s(log(DM))	3.5	279.99	879	0.224
DD ~ S(DM) + s(Subplot,bs = “re”)	51.3	−596.2	3	0.715
DD ~ s(log(DM)) + s(Subplot,bs = “re”)	50.1	−599.1	0	0.716

Abbreviations: Anom, local dandelion anomaly; Col, probability of establishment of a new vole colony; Cover, percentage cover of surface indices in March 2022; DD, dandelion depletion rate; DGR, dandelion growth rate between 2021 and 2022; DM, distance to the mounds; *D*_q_, average dandelion density in the quadrat; *D*_t_, dandelion density in the tile; *Q*, quadrat identifier; Reuse, probability of colony reuse; Subplot, subplot identifier.

**FIGURE 4:**
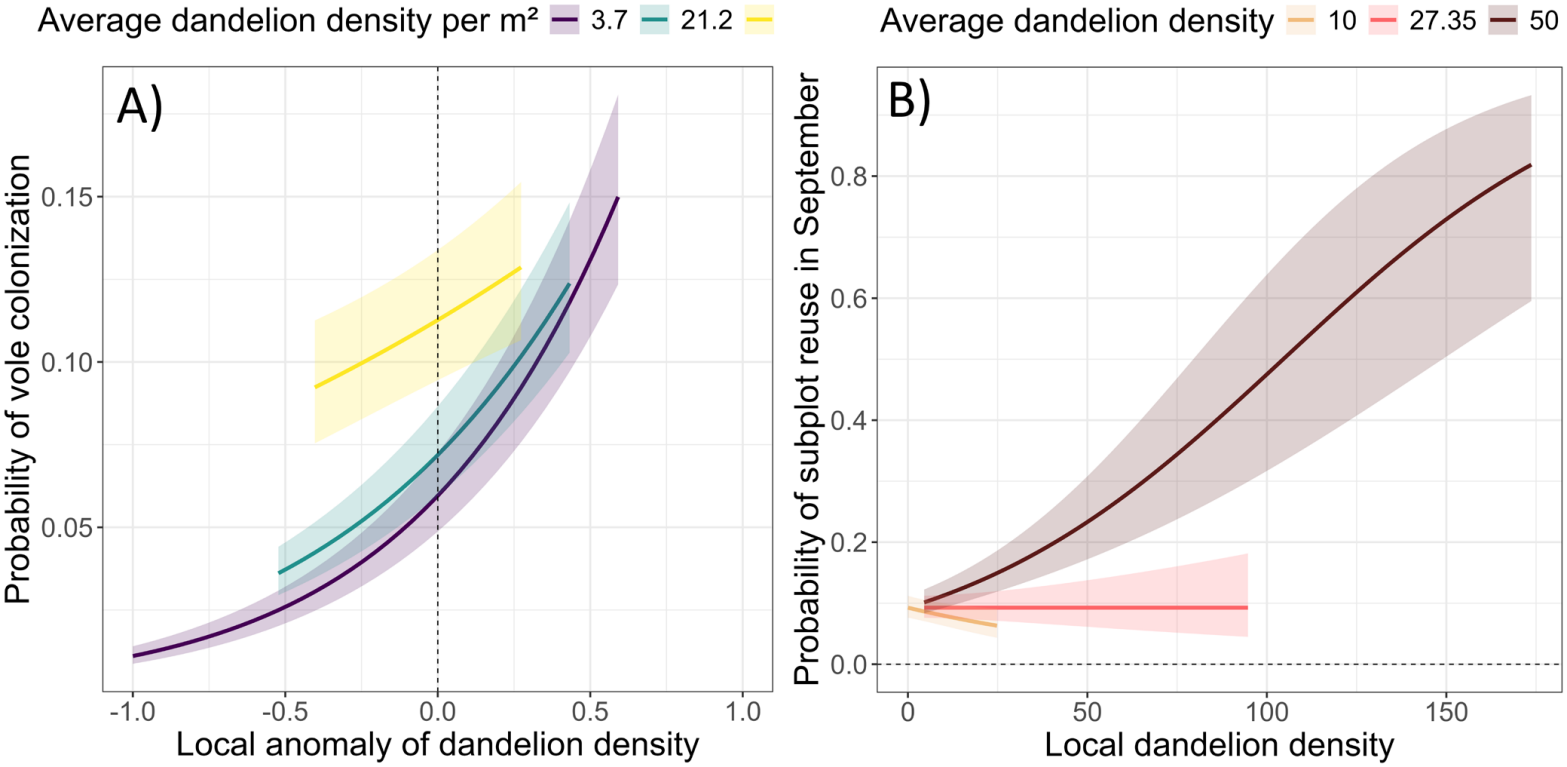
Prediction of the probability that (a) an empty tile will be colonised by voles based on dandelion anomaly (the three curves correspond to the predictions for the Q10, Q50, and Q90 quantiles of the plot dandelion density) and (b) a vole colony will be reused according to local dandelion density (the three curves represent the predictions for the Q10, the value of the transition threshold, and Q90 quantiles of the tiles dandelion density). Predictions were generated from 1 to 99 percentiles of the observed dandelion anomaly.

The probability of colonisation for a tile was significantly dependent on the average density of dandelions in the quadrat (coefficient = 0.0115 ± 0.0008, *p* < 2e‐16) and the local dandelion anomaly (coefficient = 1.803 ± 0.065, *p* < 2e‐16). However, the interaction between the dandelion anomaly and the average density of dandelions was significantly negative (coefficient = −0.0195 ± 0.0026, *p* = 2.57e‐14). This interaction means that the relationship between dandelion anomalies and colonisation depends on the average number of dandelions in the field plot. When there are plenty of dandelions in the field plot, adding or removing them locally does not significantly affect the colonisation rate. Conversely, when dandelion density is low or medium, anomalies are important (Figure 4a). In quadrats with less than four heads of dandelion flowers per m^2^ (corresponding to the 10% quantile of tile dandelion density), the probability of colonisation increased from 0.01 for tiles without dandelion to 0.13 for tiles with an anomaly of 0.5 (i.e., 5 times the quadrat average). However, in quadrats with high dandelion density (64.2 flower heads per m^2^, corresponding to the 90% quantile of tile dandelion density), the probability of colonisation only changed from 0.08 to 0.14 when the anomaly varied between −0.5 and 0.5.

### 3.3 Q2: Does the Reuse of an Old Territory Depend on Its Food Quality?

In our study, 7851 tiles were colonized in October 2021 and March 2022, but only 2785 of these tiles remained colonized in October 2022 (i.e., 35% of the burrows were reused). The model with the lowest AIC included the dandelion density within the tile and its interaction with the average dandelion density when the (fixed) quadrat effect was considered (Table 1B).

According to this model, the probability of reuse for a vole colony was significantly dependent on the density of dandelion within the tile (coefficient = −0.0263 ± 0.0088, *p* = 0.002621). However, the interaction between the tile dandelion density and the average dandelion density was highly significant (*p* = 1.12e‐06), indicating that the effect of the local dandelion density strongly depends on the average dandelion density at the quadrat level (Figure 4b). The effect was positive only in quadrats with high density of dandelion (corresponding to the 90% quantile of plot dandelion density) and null when the density was around 28 flower heads per m^2^.

### 3.4 Q3: Can a Reduction in Resources Be Observed After Territory Colonisation?

#### 3.4.1 Broad‐Scale Pattern

**FIGURE 5:**
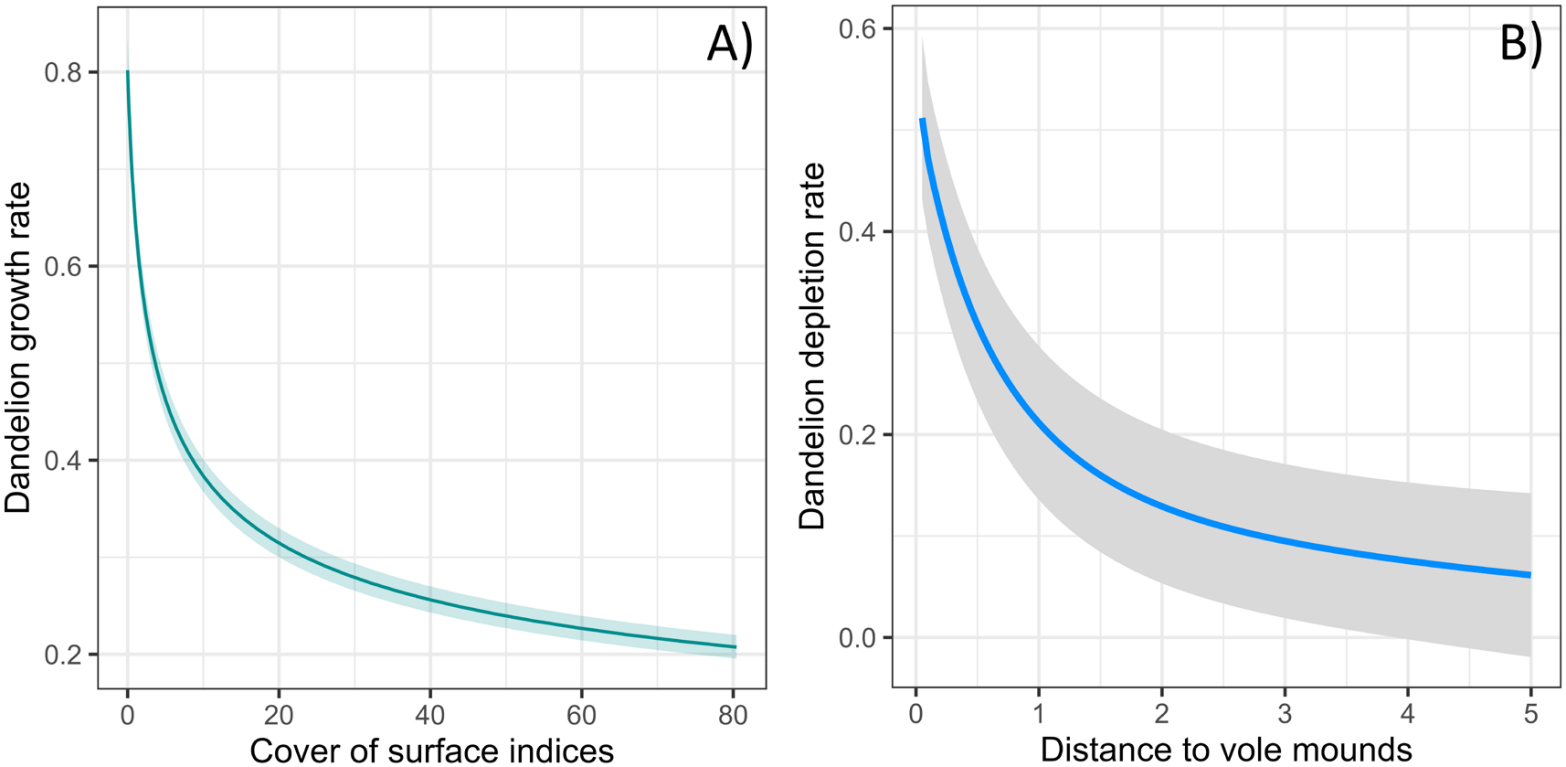
(a) Prediction of dandelion growth rate between May 2021 and May 2022 based on the cover of the surface indices of vole density in March 2022. (b) Prediction of dandelion flower‐head depletion rate as a function of distance to vole mounds, using generalised additive models (GAM).

The best model explaining DPGR selected a significant negative effect of the logarithmic transformation of vole density in March (Table 1C). A significant decrease in dandelion density was observed in areas with higher vole mound densities (coefficient = −0.3074 ± 0.0124, *p* = 2 10^−16^) (Figure 5a). The goodness‐of‐fit test for the GAM model indicates an adequate fit (P of the K check = 0.29).

#### 3.4.2 Fine‐Scale Pattern

The density of Dandelion was analyzed as a function of distance from the mounds. The logarithmic transformation distance to the earth mounds was selected as a strong fixed effect (*p* = 2 × 10^−16^) in the best model with the colony identifier included as a random effect (Table 1D). The results indicated that the density of dandelions was lower near the mounds and increased with distance from them (illustrated example in Appendix S5). This effect was measurable up to 4 m around the mounds (Figure 5b).

## 4 Discussion

Our study investigated the selection of water vole habitat in relation to the food quality of the territories. We also examined the feedback of vole colonization (after habitat selection) on the food quality of the territory. To achieve this, we used an unmanned aerial vehicle (UAV, i.e., drone).

### 4.1 Methodological Approach: Use of Drone to Investigate Plant‐Vole Interactions

Many scientists have developed methods for detecting animal densities using UAV photography, provided the animals are not cryptic from the air. UAVs have been used to detect wetland birds such as flamingos (Valle 2022), seagulls (Corregidor‐Castro et al. 2021), or geese (Aniceto et al. 2018). Regarding rodents, studies have shown that various burrowing species such as marmots (Enkhbat et al. 2023), prairie dogs (Kearney et al. 2023), and ground squirrels (Gedeon et al. 2022) can also be tracked using UAVs. Detecting the densities of water voles from earth mounds is therefore not a major novelty, although it is useful for quantifying populations or assessing damage. Numerous studies have also focused on the detection of particular plant species such as endangered dwarf bear poppy (Rominger and Meyer 2019), cultivated rice (Zheng et al. 2020), or ornamental plants (Bayraktar et al. 2020). The great advantage of UAVs is their ability to survey large areas over time, allowing detailed temporal analyses. Therefore, our study is innovative, as it demonstrates the possibility of detecting two different species and inferring their relationships through the spatial structure of these two species across time.

A key point in our approach was the unpredictability of vole settlement locations, making it essential to monitor large areas. Placing the study in the favorable context of population dynamics (i.e., the growth phase) also greatly improved the probability of observing colonizations.

Drone photography requires specific environmental conditions that could be difficult to meet. There must be no wind or rain, and the lighting must be at least calibrated. In our case, calm weather conditions free of wind and rain were particularly scarce in mountain climates. Photographing dandelions with drones added further complexity due to their brief and synchronized flowering period (5–10 days), which is influenced by meteorological factors (the opening of dandelion flowers is temperature dependent, requiring a minimum of 13°C Tanaka et al. 1988) and elevation. These constraints limited the area we monitored. Additionally, post‐processing and interpretation of drone imagery are time‐consuming and require substantial data storage capacity. In our case, as the objects we detected (brown spots and yellow dots in a sea of uniform green) were relatively simple, we used simple detection algorithms. However, with more complex objects, it is possible to develop more technical methods such as deep learning.

We also believe that this technique could provide useful information for habitat selection in rodents. Some candidate species, such as the common vole, which lives in areas where vegetation is short and whose tunnel mouths can be spotted, seem more realistic than others, such as field mice, tree squirrels, or lemmings, which are more cryptic and live in more complex environments, as seen from an aerial photograph.

### 4.2 Habitat Selection on Food Availability

In this study, we were interested in how the quality of a habitat type, grassland, is perceived and selected by water voles. To achieve this, we focused on very low‐contrast environments and avoided different habitat types, such as hedges, forests, and wetlands. We concentrated on the species' preferred habitat, where we observed the highest densities (Morilhat et al. 2007). We also avoided variations in grass height between the monitored areas, as this factor is known to modulate the risk of predation for voles (Getz 1985; see, e.g., Jacob 2003). This approach allowed us to establish a quasi‐experimental framework with food availability being the only variable in our study. However, our methodology is relatively well‐suited to areas where water vole outbreaks occur, typically large open fields of grassland largely devoid of trees.

Rodents are well known to have habitat preferences (see, e.g., Bonnet et al. 2013; Heroldová et al. 2021) or to forage in selected patches (see, e.g., Kopp 1988). High‐quality food has been shown to provide significant benefits for voles at the individual scale (e.g., Batzli 1986) and to permit high population densities (e.g., Hall et al. 1991) or to prevent winter population crashes (e.g., Johnsen et al. 2017). Furthermore, many studies have shown that voles are able to overgraze in certain cases (e.g., Huitu et al. 2003). Although several studies have focused on the effect of the departure habitat on dispersal (e.g., Lin et al. 2006; Rémy et al. 2011), few have examined the arrival habitat probably because of methodological problems (Glorvigen et al. 2013). However, experimental evidence suggests, consistent with our findings, that voles preferentially settle in habitats with higher food quality, among other selection criteria (Glorvigen et al. 2013).

The originality of our study lies in demonstrating that food quality is a strong driver for the selection of water vole habitats in natural settings, probably relegating predation to a secondary role, at least during the growth phase. Water voles have high antipredatory behaviors (see model species paragraph), suggesting that predation has been a significant force of selection. However, during the growth phase, high‐quality habitats are common and probably easy to find. Dispersal distances are also probably short, even to locate good‐quality habitats, thus eliminating the trade‐off between food quality and predation risk. Finally, the degradation of habitat quality upon colonization may have significant implications for individual replacement and thus on population dynamics.

### 4.3 Possible Consequences on Population Dynamics and Cycle Theory

In this study, we focus primarily on the scale of vole colonies. By monitoring large areas several times over a 2‐year period, we were able to retrospectively characterize vole territories prior to colonization. We considered dandelions to be an important component of the trophic quality of the water vole's habitat due to its winter storage behavior (Lisse et al. 2024). On the territory scale, we observed that dandelion density is a factor of attractiveness. We also measured that once a territory is occupied, dandelion densities decrease, making the territory less likely to be recolonized. The quality of the habitat and, therefore, its attractiveness, can only be restored when the dandelion population regenerates sufficiently, which is suspected to take considerable time.

At the plot level, we observed that vole growth rates are higher if the dandelion density is high. Therefore, we expect delayed negative feedback on dandelion densities, making plots less attractive. This process can theoretically be transferred to the landscape scale if dandelions' densities synchronize at high levels during the phase of low vole density.

Changes in the dispersal rate of young voles necessarily impact population structure and genetics. Cerqueira et al. (2006) showed that during the decline, voles continue to reproduce but there is a deficit in the number of young individuals. Berthier et al. (2014) observed that dispersal distances are relatively low at the start of the growth phase, highlighting close‐to‐home colonization, and increase during the peak. Finally, Giraudoux et al. (1997) observed that outbreaks begin in epicenters and then move in a traveling wave phenomenon. These massive movements in density throughout the landscape can only be explained by extensive dispersal. These three results on the same species model under comparable conditions, obtained at different scales, are all consistent with strong interdependent plant‐herbivore relationships mediated by the dispersal of young voles.

Four main types of rodent‐plant interactions may be responsible for cyclical fluctuations (Soininen and Neby 2023): (1) intrinsic cyclical quality/quantity of plants, (2) reduction in the quantity of available resources through overgrazing, (3) plants' defenses in response to grazing induce a decrease in the quality of preferred plants, (4) reduction in quality through a change in diet in favor of elements of lower quality. Our results may be consistent with the second hypothesis as well as with the fourth. This depends on how habitat selection varies with density, which we did not measure in this study. However, to determine whether dandelions are a fundamental parameter of water vole cycles, we need to measure whether vole and dandelion densities covary throughout an entire cycle. In conclusion, our study shows that voles select their habitat based on the quality of its food. It also demonstrates that, once voles colonize an area, the quality of the food available there decreases, rendering it unsuitable for new voles to settle there. This feedback loop exists because fossorial water voles depend heavily on a specific resource. Even if it may seem obvious, it highlights the importance of thoroughly understanding the vole diet in the study area when investigating the plant hypothesis.

## Author Contributions

**Marion Buronfosse:** data curation (supporting), formal analysis (lead), investigation (supporting), methodology (supporting), resources (supporting), software (lead), visualization (lead), writing – original draft (lead). **Hélène Lisse:** conceptualization (lead), data curation (lead), project administration (lead), resources (lead), software (supporting), writing – original draft (supporting), writing – review and editing (supporting). **Geoffroy Couval:** data curation (supporting). **Aurélien Levret:** data curation (supporting). **François Gillet:** formal analysis (supporting), software (supporting), validation (supporting), visualization (supporting), writing – original draft (supporting). **Virginie Lattard:** conceptualization (supporting), funding acquisition (lead), methodology (supporting), resources (supporting), writing – original draft (supporting), writing – review and editing (supporting). **Adrien Pinot:** conceptualization (lead), formal analysis (lead), funding acquisition (lead), investigation (supporting), methodology (lead), project administration (supporting), resources (supporting), software (lead), supervision (lead), validation (lead), visualization (supporting), writing – original draft (lead), writing – review and editing (lead).

## Conflicts of Interest

The authors declare no conflicts of interest.

## Supporting information


**Data S1:** ece372208‐sup‐0001‐Supinfo.docx.

## Data Availability

The metadata, data, and code used for this study (formatted with the recommendations of Jenkins et al. 2023) are available at the following link: https://doi.org/10.5061/dryad.8w9ghx3xs.

## References

[ece372208-bib-0001] Airoldi, J. P. 1976. “Expériences de Capture et Recapture Chez le Campagnol Terrestre, *Arvicola terrestris* Scherman Shaw (Mammalia‐Rodentia).” Revue d'Écologie 1: 31–51.

[ece372208-bib-0002] Airoldi, J.‐P. , and D. D. Werra . 1993. “The Burrow System of the Fossorial Form of the Water Vole (*Arvicola terrestris* Scherman Shaw.) (Mammalia, Rodentia): An Approach Using Graph Theoretical Methods and Simulation Models.” 57, no. 3: 423–434.

[ece372208-bib-0003] Aniceto, A. S. , M. Biuw , U. Lindstrøm , S. A. Solbø , F. Broms , and J. Carroll . 2018. “Monitoring Marine Mammals Using Unmanned Aerial Vehicles: Quantifying Detection Certainty.” Ecosphere 9, no. 3: e02122.

[ece372208-bib-0004] Batzli, G. O. 1986. “Nutritional Ecology of the California Vole: Effects of Food Quality on Reproduction.” Ecology 67: 406–412.

[ece372208-bib-0005] Bayraktar, E. , M. E. Basarkan , and N. Celebi . 2020. “A Low‐Cost UAV Framework Towards Ornamental Plant Detection and Counting in the Wild.” ISPRS Journal of Photogrammetry and Remote Sensing 167: 1–11.

[ece372208-bib-0006] Berthier, K. , S. Piry , J. F. Cosson , et al. 2014. “Dispersal, Landscape and Travelling Waves in Cyclic Vole Populations.” Ecology Letters 17, no. 1: 53–64. 24237964 10.1111/ele.12207

[ece372208-bib-0007] Bonnet, T. , L. Crespin , A. Pinot , L. Bruneteau , V. Bretagnolle , and B. Gauffre . 2013. “How the Common Vole Copes With Modern Farming: Insights From a Capture–Mark–Recapture Experiment.” Agriculture, Ecosystems & Environment 177: 21–27.

[ece372208-bib-0008] Burnham, K. P. , and D. R. Anderson . 2002. Model Selection and Multimodel Inference. 2nd ed. Springer.

[ece372208-bib-0009] Cerqueira, D. , B. De Sousa , C. Gabrion , P. Giraudoux , J. P. Quéré , and P. Delattre . 2006. “Cyclic Changes in the Population Structure and Reproductive Pattern of the Water Vole, *Arvicola terrestris* Linnaeus, 1758.” Mammalian Biology 71, no. 4: 193–202.

[ece372208-bib-0010] Cole, F. R. , and G. O. Batzli . 1979. “Nutrition and Population Dynamics of the Prairie Vole, *Microtus ochrogaster* , in Central Illinois.” Journal of Animal Ecology 48: 455–470.

[ece372208-bib-0011] Corregidor‐Castro, A. , T. E. Holm , and T. Bregnballe . 2021. “Counting Breeding Gulls With Unmanned Aerial Vehicles: Camera Quality and Flying Height Affects Precision of a Semi‐Automatic Counting Method.” Ornis Fennica 98, no. 1: 33–45.

[ece372208-bib-0012] Crego, R. D. , J. E. Jiménez , and R. Rozzi . 2018. “Macro‐and micro‐Habitat Selection of Small Rodents and Their Predation Risk Perception Under a Novel Invasive Predator at the Southern End of the Americas.” Mammal Research 63: 267–275.

[ece372208-bib-0013] DeCesare, N. J. , M. Hebblewhite , M. Bradley , D. Hervieux , L. Neufeld , and M. Musiani . 2014. “Linking Habitat Selection and Predation Risk to Spatial Variation in Survival.” Journal of Animal Ecology 83, no. 2: 343–352. 24099266 10.1111/1365-2656.12144 PMC4285818

[ece372208-bib-0014] Delattre, P. , R. Clarac , J. P. Melis , D. R. J. Pleydell , and P. Giraudoux . 2006. “How Moles Contribute to Colonization Success of Water Voles in Grassland: Implications for Control.” Journal of Applied Ecology 43, no. 2: 353–359.

[ece372208-bib-0015] Enkhbat, E. , U. U. Bayanmunkh , A. Yunden , et al. 2023. “Use of Drone Technology to Monitor and Map Endangered Marmot Populations in Mongolian Grasslands.” Hystrix, the Italian Journal of Mammalogy 34, no. 1: 62–67.

[ece372208-bib-0016] Franklin, A. B. , D. R. Anderson , R. J. Gutiérrez , and K. P. Burnham . 2000. “Climate, Habitat Quality, and Fitness in Northern Spotted Owl Populations in Northwestern California.” Ecological Monographs 70, no. 4: 539–590.

[ece372208-bib-0017] Fretwell, S. D. 1972. Populations in Seasonal Environments. Princeton University Press.

[ece372208-bib-0018] Fretwell, S. D. , and H. L. Lucas . 1970. “On Territorial Behavior and Other Factors Influencing Habitat Distribution in Birds.” Acta Biotheoretica 19, no. 1: 16–36.

[ece372208-bib-0019] Gaillard, J. M. , M. Hebblewhite , A. Loison , et al. 2010. “Habitat–Performance Relationships: Finding the Right Metric at a Given Spatial Scale.” Philosophical Transactions of the Royal Society, B: Biological Sciences 365, no. 1550: 2255–2265. 10.1098/rstb.2010.0085 PMC2894964 20566502

[ece372208-bib-0020] Gedeon, C. I. , M. Árvai , G. Szatmári , et al. 2022. “Identification and Counting of European Souslik Burrows From UAV Images by Pixel‐Based Image Analysis and Random Forest Classification: A Simple, Semi‐Automated, Yet Accurate Method for Estimating Population Size.” Remote Sensing 14, no. 9: 2025.

[ece372208-bib-0021] Getz, L. L. 1985. “Habitats.” In Biology of New World Microtus. Special Publication No. 8, edited by R. H. Tamarin , 286–309. American Society of Mammalogists.

[ece372208-bib-0022] Giraudoux, P. , P. Delattre , M. Habert , et al. 1997. “Population Dynamics of Fossorial Water Vole ( *Arvicola terrestris* Scherman): A Land Use and Landscape Perspective.” Agriculture, Ecosystems & Environment 66, no. 1: 47–60.

[ece372208-bib-0023] Giraudoux, P. , B. Pradier , P. Delattre , S. Deblay , D. Salvi , and R. Defaut . 1995. “Estimation of Water Vole Abundance by Using Surface Indices.” Acta Theriologica 40, no. 1: 77–96.

[ece372208-bib-0024] Glorvigen, P. , G. Gundersen , H. P. Andreassen , and R. A. Ims . 2013. “The Role of Colonization in the Dynamics of Patchy Populations of a Cyclic Vole Species.” Oecologia 173: 161–167. 23443355 10.1007/s00442-013-2614-y

[ece372208-bib-0025] Hall, A. T. , P. E. Woods , and G. W. Barrett . 1991. “Population Dynamics of the Meadow Vole ( *Microtus pennsylvanicus* ) in Nutrient‐Enriched Old‐Field Communities.” Journal of Mammalogy 72: 332–342.

[ece372208-bib-0026] Heroldová, M. , J. Šipoš , J. Suchomel , and J. Zejda . 2021. “Influence of Crop Type on Common Vole Abundance in Central European Agroecosystems.” Agriculture, Ecosystems & Environment 315: 107443.

[ece372208-bib-0027] Huitu, O. , M. Koivula , E. Korpimäki , T. Klemola , and K. Norrdahl . 2003. “Winter Food Supply Limits Growth of Northern Vole Populations in the Absence of Predation.” Ecology 84, no. 8: 2108–2118.

[ece372208-bib-0028] Hutchinson, G. E. 1957. “Concluding Remarks.” Cold Spring Harbor Symposia on Quantitative Biology 22: 415–427.

[ece372208-bib-0029] Hutto, R. 1985. “Habitat Selection by Nonbreeding, Migratory Land Birds.” In Habitat Selection in Birds, 455–476. Academic Press, Inc.

[ece372208-bib-0030] Ims, R. A. , and H. P. Andreassen . 2000. “Spatial Synchronization of Vole Population Dynamics by Predatory Birds.” Nature 408, no. 6809: 194–196. 11089971 10.1038/35041562

[ece372208-bib-0031] Jacob, J. 2003. “Short‐Term Effects of Farming Practices on Populations of Common Voles.” Agriculture, Ecosystems & Environment 95, no. 1: 321–325.

[ece372208-bib-0032] Jenkins, G. B. , A. P. Beckerman , C. Bellard , et al. 2023. “Reproducibility in Ecology and Evolution: Minimum Standards for Data and Code.” Ecology and Evolution 13, no. 5: e9961. 37181203 10.1002/ece3.9961 PMC10170304

[ece372208-bib-0033] Johnsen, K. , R. Boonstra , S. Boutin , O. Devineau , C. J. Krebs , and H. P. Andreassen . 2017. “Surviving Winter: Food, but Not Habitat Structure, Prevents Crashes in Cyclic Vole Populations.” Ecology and Evolution 7, no. 1: 115–124. 28070280 10.1002/ece3.2635 PMC5216623

[ece372208-bib-0034] Kearney, S. P. , L. M. Porensky , D. J. Augustine , and D. W. Pellatz . 2023. “Toward Broad‐Scale Mapping and Characterization of Prairie Dog Colonies From Airborne Imagery Using Deep Learning.” Ecological Indicators 154: 110684.

[ece372208-bib-0035] Kopp, R. 1988. “Les Choix Alimentaires de la Forme Fouisseuse du Campagnol Terrestre ( *Arvicola terrestris* Scherman): Essais en Terrarium.” Bulletin EOPP 18: 393–400.

[ece372208-bib-0036] Lin, Y. K. , B. Keane , A. Isenhour , and N. G. Solomon . 2006. “Effects of Patch Quality on Dispersal and Social Organization of Prairie Voles: An Experimental Approach.” Journal of Mammalogy 87, no. 3: 446–453.

[ece372208-bib-0037] Lisse, H. , M. Buronfosse , C. Jacquet , et al. 2024. “Is Water Vole Diet Consistent With the Plant Hypothesis for Explaining Population Fluctuations?” *bioRxiv*.

[ece372208-bib-0038] Lisse, H. , M. Buronfosse , G. Sobczyck‐Moran , et al. 2022. “Biologie du Campagnol Terrestre en Auvergne.” Phytoma, no. 754: 30–34.

[ece372208-bib-0039] Lisse, H. , and A. Pinot . 2024. “A New Camera‐Trapping Device, the Campascope, to Study Feeding Behaviour of Subterranean Rodents.” Mammal Research 69, no. 2: 303–311.

[ece372208-bib-0040] McNab, B. K. 1983. “Energetics, Body Size, and the Limits to Endothermy.” Journal of Zoology 199, no. 1: 1–29.

[ece372208-bib-0041] Morilhat, C. , N. Bernard , C. Bournais , C. Meyer , C. Lamboley , and P. Giraudoux . 2007. “Responses of *Arvicola terrestris* Scherman Populations to Agricultural Practices, and to *Talpa europaea* Abundance in Eastern France.” Agriculture, Ecosystems & Environment 122, no. 3: 392–398.

[ece372208-bib-0042] Morris, D. W. 2003. “Toward an Ecological Synthesis: A Case for Habitat Selection.” Oecologia 136: 1–13. 12690550 10.1007/s00442-003-1241-4

[ece372208-bib-0043] Pascal, M. , and T. Boujard . 1987. “Essai de Typologie de Paramètres Démographiques et Morphologiques de la Fraction Colonisatrice d'Une Population de Campagnols Terrestres ( *Arvicola terrestris* Scherman (Shaw.)).” Revue d'Écologie (La Terre et la Vie) 42, no. 4: 357–376.

[ece372208-bib-0044] Potapov, M. , V. Rogov , L. Ovchinnikova , et al. 2004. “The Effect of Winter Food Stores on Body Mass and Winter Survival of Water Voles, *Arvicola terrestris* , in Western Siberia: The Implications for Population Dynamics.” Folia Zoologica‐Praha 53: 37–46.

[ece372208-bib-0045] Pulliam, H. R. 2000. “On the Relationship Between Niche and Distribution.” Ecology Letters 3, no. 4: 349–361.

[ece372208-bib-0046] Rémy, A. , J.‐F. Le Galliard , G. Gundersen , H. Steen , and H. P. Andreassen . 2011. “Effects of Individual Condition and Habitat Quality on Natal Dispersal Behaviour in a Small Rodent.” Journal of Animal Ecology 80, no. 5: 929–937. 21521215 10.1111/j.1365-2656.2011.01849.x

[ece372208-bib-0047] Rominger, K. , and S. E. Meyer . 2019. “Application of UAV‐Based Methodology for Census of an Endangered Plant Species in a Fragile Habitat.” Remote Sensing 11, no. 6: 719.

[ece372208-bib-0048] Saucy, F. 1994. “Density Dependence in Time Series of the Fossorial Form of the Water Vole, *Arvicola terrestris* .” Oikos 71: 381–392.

[ece372208-bib-0049] Saucy, F. , and B. Schneiter . 1997. “Juvenile Dispersal in the Vole *Arvicola terrestris* During Rainy Nights: A Preliminary Report.” Bulletin de la Societe Vaudoise des Sciences Naturelles 84, no. 4: 333–345.

[ece372208-bib-0050] Soininen, E. , and M. Neby . 2023. “Small Rodent Population Cycles and Plants‐After 70 Years, Where Do We Go?” Biological Reviews 99: 265–294. 37827522 10.1111/brv.13021

[ece372208-bib-0051] Tanaka, O. , Y. Tanaka , and H. Wada . 1988. “Photonastic and Thermonastic Opening of Capitulum in Dandelion, Taraxacum Otficinale and Taraxacum Japonicum.” Botanical Magasine Tokyo 101: 103–110.

[ece372208-bib-0052] Valle, R. G. 2022. “Rapid Drone Semi‐Automated Counts of Wintering Greater Flamingos ( *Phoenicopterus roseus* ) as a Tool for Amateur Researchers.” Ibis 164, no. 1: 320–328.

[ece372208-bib-0101] Yoccoz, N. G. , and R. A. Ims . 1999. “Demography of Small Mammals in Cold Regions: The Importance of Environmental Variability.” Ecological Bulletins 47: 137–144.

[ece372208-bib-0054] Zheng, H. , X. Zhou , J. He , et al. 2020. “Early Season Detection of Rice Plants Using RGB, NIR‐GB and Multispectral Images From Unmanned Aerial Vehicle (UAV).” Computers and Electronics in Agriculture 169: 105223.

[ece372208-bib-0055] Zub, K. , Z. Borowski , P. A. Szafrańska , M. Wieczorek , and M. Konarzewski . 2014. “Lower Body Mass and Higher Metabolic Rate Enhance Winter Survival in Root Voles, *Microtus oeconomus* .” Biological Journal of the Linnean Society 113, no. 1: 297–309.

[ece372208-bib-0056] Zynel, C. A. , and B. A. Wunder . 2002. “Limits to Food Intake by the Prairie Vole: Effects of Time for Digestion.” Functional Ecology 16, no. 1: 58–66.

