# Supplementary material for: Fossorial Form of Water Voles Select and Overexploit High‐Quality Habitats, Hindering Future Colonizations Evidence From Drone‐Based Monitoring of Dandelion‐Vole Interactions in Mountain Meadows
Source: Ecol Evol. 2025 Sep 22;15(9):e72208. doi: 10.1002/ece3.72208 (PMC12453612; doi:10.1002/ece3.72208)
Supplement: Supplementary file 1 — Data S1: ece372208‐sup‐0001‐Supinfo.docx. [file ECE3-15-e72208-s001.docx]

# Supporting information

## **SuppInfo 1 – METHODS: Study area and field plot selection**

This work was made by Hélène Lisse and Adrien Pinot

**First phase: determine phase of cycle using Arvicola Obs**

The ArvicolaObs application is used to record vole densities twice a year (spring/autumn) across the Massif Central at a landscape scale (<https://www.arvicola-obs.fr/>, Michelin *et al.* 2025). To achieve this, ‘observational points’ are located approximately every kilometer in areas known to experience vole outbreaks. Observers visit these points and provide a score for the average vole density within a 60-meter radius of the point. These scores range from 0 (no voles) to 5 (extreme outbreak). This tool was introduced in 2017.

While the tool is not perfect (as information is not collected with the same spatial coverage each year) it allows for the observation of landscape dynamics, as can be seen in the following figure.


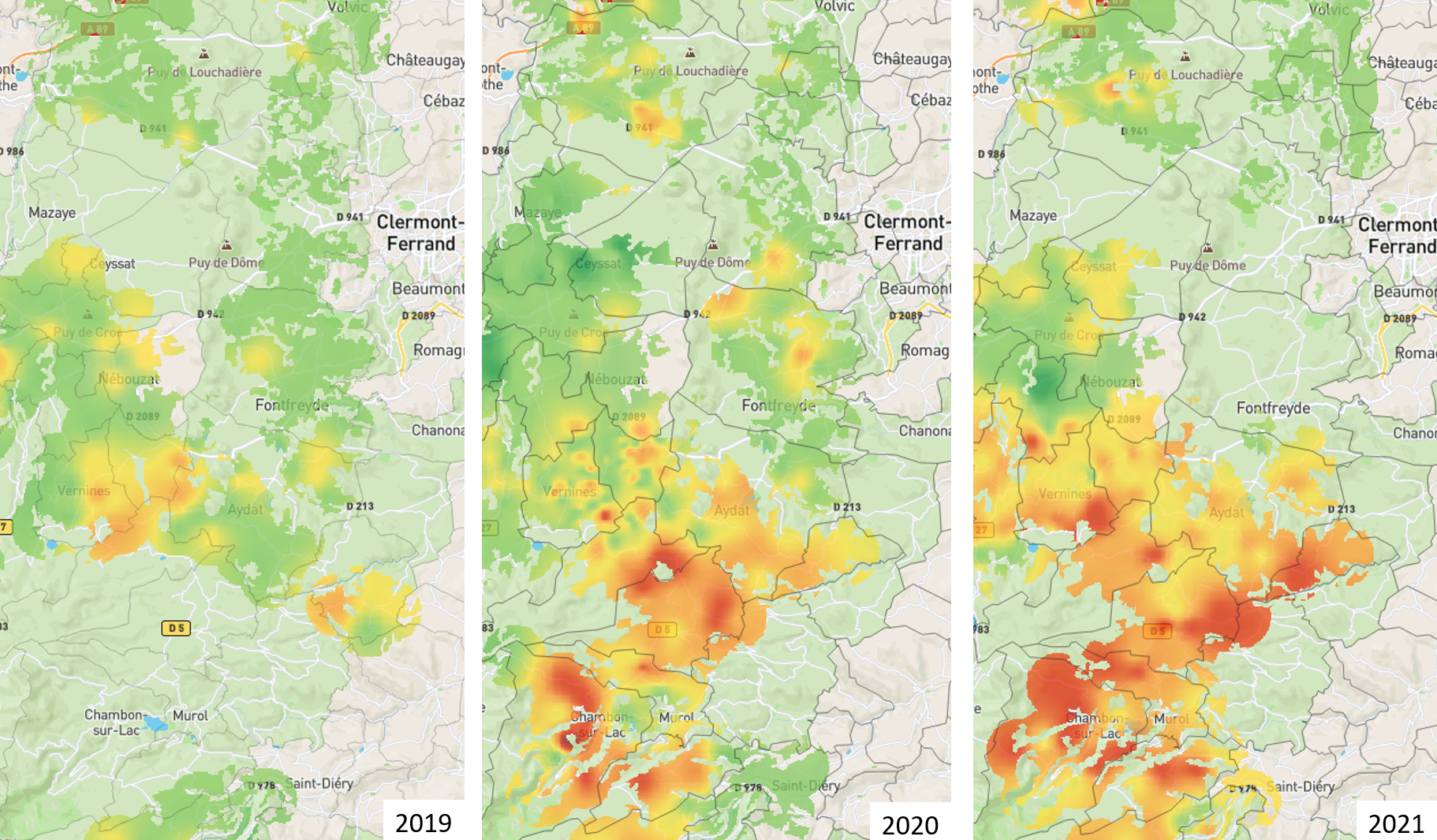


Figure 1: Vole densities at landscape scale. Light green represents grassland open field. Light grey represents cultures. Vole densities were estimated using kriging from the “observational points” collected through ArvicolaObs. Green areas represent low water vole densities, yellow represents moderate water vole densities and orange to red represent outbreaks ranging from moderate to severes.

We recovered these data thanks to professor Yves Michelin, the designer and promoter of the tool. We then calculated annual growth rates (from autumn to autumn) at the observational point level. Using GIS, we visually selected areas where densities increased from very low to low values, which may indicate the start of the growth phase. Based on our expertise, we focused on large open fields that appeared synchronous (> 10km²).

**Second phase: verifying the amplitude of the outbreak**

We tried to retrieve all the data on water voles that had been collected through trapping from government departments and vole population managers, in order to verify whether an outbreak was likely in the areas we had previously identified. This research revealed the occurrence of significant outbreaks in the study area during the 80s and 90s, with several locations reporting densities of over 400 voles per hectare.

**Last phase: validation in the field**

We went out into the field to check that the zones corresponded to the situation we were looking for. We compared our zones with those in the field (vole managers, researchers, farmers). This allowed us to select field plots in the growth phase.

**Definition of selected study area**

The CM area was a 11.5 by 23 km rectangle. It was located between the Puy de Dôme mountain in the north-east and the Sancy mountain in the south-west. This plateau, which has an average altitude of 972 meters above sea level, is devoted to milk and meat production. Land use was calculated using Corine Land Cover data for forests, as well as the RPG declarations that farmers make to the government in order to obtain subsidies.


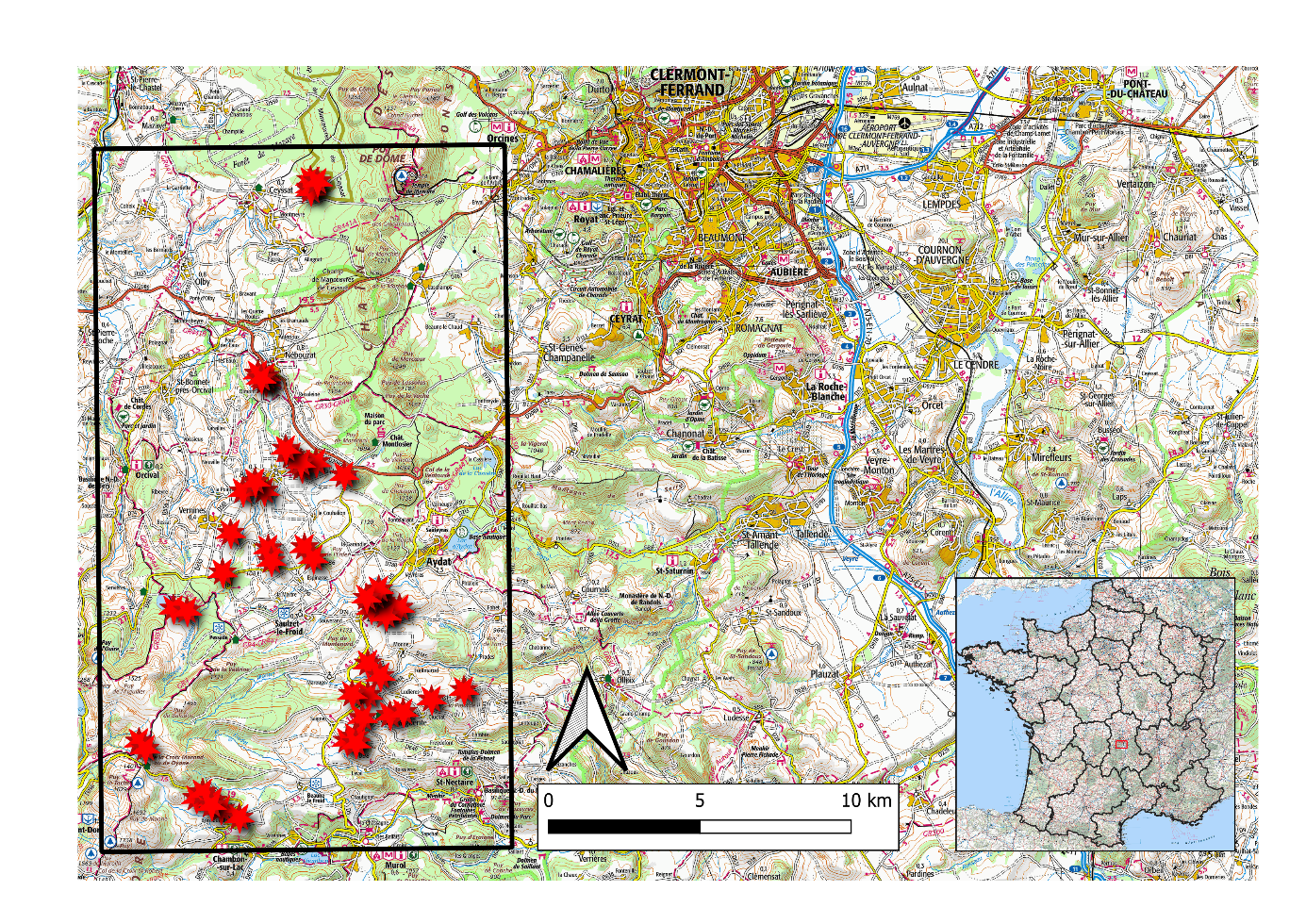


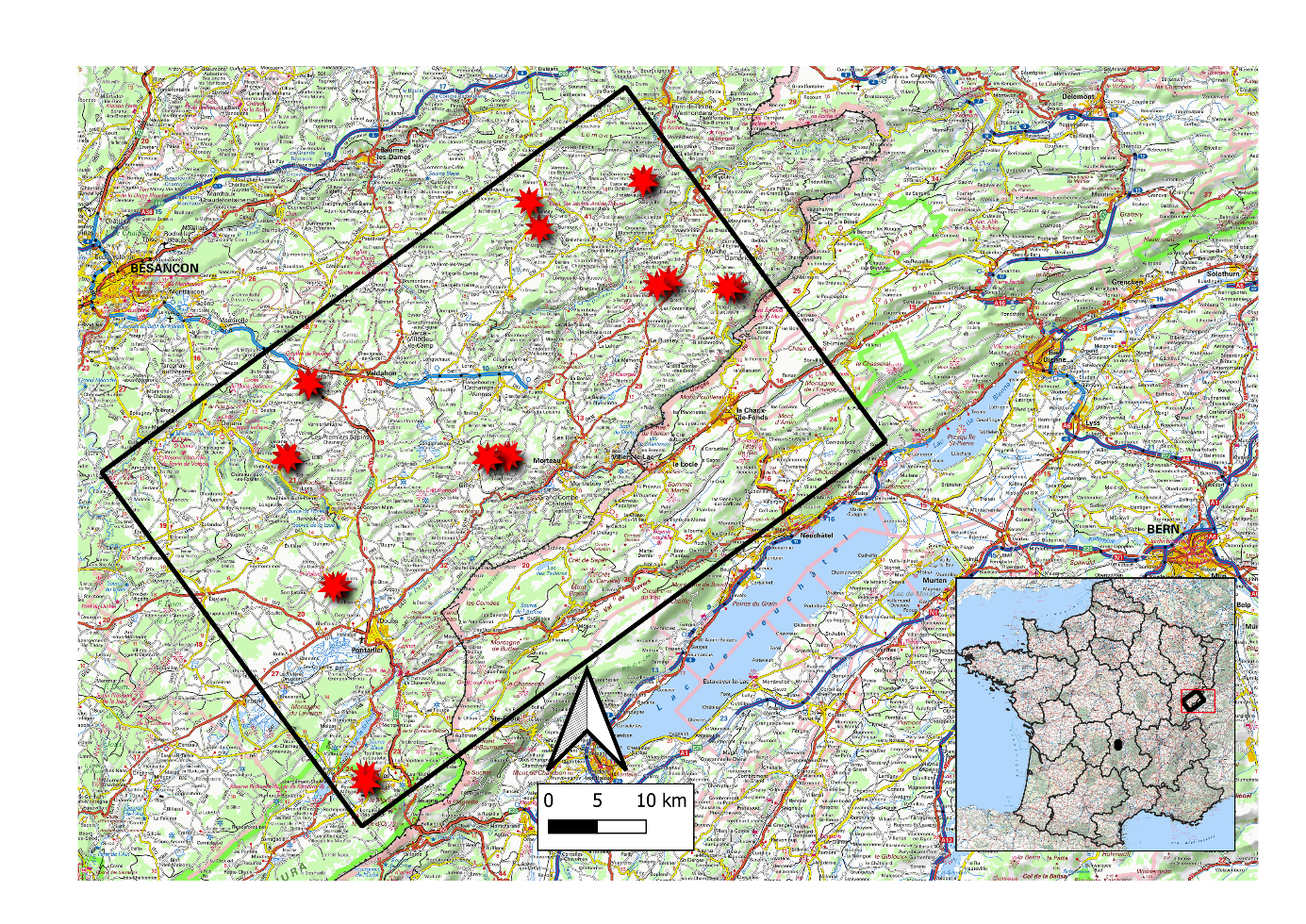


The area in Jura was a 44 by 66 km rectangle located next to Lake Neuchâtel in Switzerland. This plateau, with an average altitude of 846 meters above sea level, is devoted to milk production. Land use was calculated using Corine Land Cover data for forests and RPG declarations made by farmers to obtain subsidies, in the French part of the study area.

This study area was less focused on the vole growth phase, as we did not have access to equally precise information. Fieldwork in this area was extended primarily to generalise the model observed in the Massif Central.

*Michelin Y., Vidal S., Pinot A. (2025). Un nouveau dispositif de surveillance des campagnols terrestres, Phytoma.*

## **SuppInfo 2 – METHODS: Relation between flowers number and root biomass in dandelion**

This work was made by Hélène Lisse, Marion Buronfosse and Adrien Pinot

**Context**

In our study area, the dandelion is a key winter resource for water voles. They store the roots of more than 200 dandelions in food caches (Lisse et al., 2024). Knowing the heterogeneity of dandelion root density is therefore an important issue.

**Problematic**

We aim to determine whether the number of dandelion flowers is a reliable indicator of the root biomass available to voles.

**Methods**

We used two approaches to examine the relationship between flower number and root biomass. First, we reused the data from the conference paper by Datcu et al. (2018) to observe the relationship between the biomass of flowers and roots. As the paper included only eight data points, the analysis was limited to a visual inspection of the figure.

Secondly, during the flowering period in May, we collected a large number of dandelions in our CM study area. A sample of 15 dandelions was collected from each of 10 different field plots along a fertility gradient (as observed using NDVI satellite data from Copernicus, from 1344 to 3296 PPI.day, mean 2314.9 PPI.day – See the 'Total Productivity' section in the WEKEO Viewer for more information). All field plots were located between 880 and 950 metres above sea level and consisted of permanent grassland. The plants were collected during their flowering period in May 2022, over the course of two consecutive days. We then measured the number of flowers and root biomass in the laboratory. The samples were stored in the refrigerator before processing. A maximum of two days elapsed between field collection and measurement. We used a Poisson model to analyse the relationship between the number of flowers and root weight.

**Results**

In both cases, we visually observed a strong correlation (panel left for Dactu *et al.* data’s, panel left for our datas).


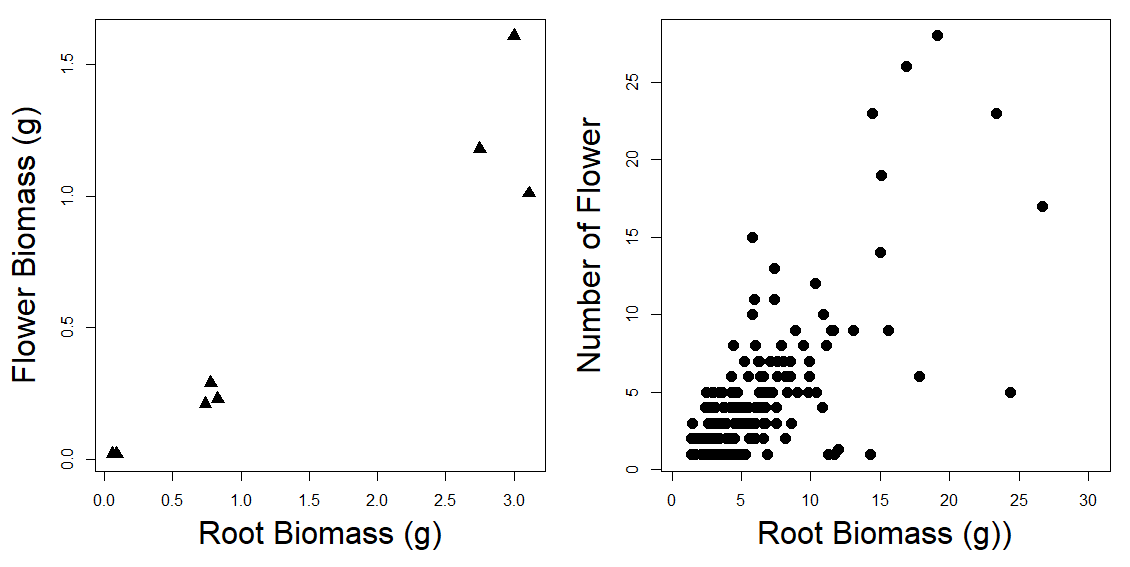


The Poisson model based on the CM study area explains 50.8% of the total deviance. A positive and highly significant relationship was found between root weight and the number of flowers.

|  | Estimate | Standard Error | P-value |
| --- | --- | --- | --- |
| Intercept | 0.907 | 0.059 | 2e-16 |
| Root weight | 0.094 | 0.005 | 2e-16 |

**Discussion**

These results show that:

1) There is a correlation between the number of flowers and root biomass in our CM study area.

2) It seems possible to generalise.

3) the same relationship is also expected in the Jura.

**References**

*Datcu AD, Tanase, RM, Inovici N. (2018) Biomass Allocation Parameters in Taraxacum officinale. International conference of life sciences.*

*Lisse, H., Buronfosse, M., Jacquet, C., Sobczyk-Moran, G., Ramadier, E., Fafournoux, A., Lattard, V., & Pinot, A. (2024). Is water vole diet consistent with the plant hypothesis for explaining population fluctuations? bioRxiv.*

##

## **SuppInfo 3 - METHODS: Remote sensing protocol**

This work was made by Marion Buronfosse, Hélène Lisse and Adrien Pinot

The drone was an Anaphi Parrot (320 g), programmable and equipped with a standard RGB sensor (sensor size: 1/2.4’’ CMOS, 5344x4016 pixels). The drone flight was programmed on each quadrat with Pix4d capture (flight parameters: altitude 30 m, speed 2 m/s, image coverage 80%). To monitor the 4800 m², the flight took about 10 minutes and produced 120 images. The images were merged and georeferenced using Pix4d Field program.

Each image is composed of the three bands in the visible spectrum: the red, the green and the blue. The color ranges of the bands were used to isolate the elements of interest from the rest of the image (e.g. deep yellow corresponds to R = 1, G = 1, B = 0). In our case, we focused on the brown of the soil pull up by the digging activities of voles, and the yellow of the dandelion flower heads.

To analyze the images, we used the countcolor R package developed by Hannah Weller (Hooper et al., 2020). To improve the accuracy of the mound detection, we converted the images to false color by transforming the red band. The red band was replaced by the ExGR indices, which was originally designed to highlight vegetation, and distinguish the bare soil (Meyer & Neto, 2008).

ExGR = 3 × φgreen – 2.4 × φred – φblue

with φgreen the green band, φred the red one and φblue the blue one.

The bare soil has different hue depending on the exposure of the plot when the image was taken, or on the natural color of the soil. Therefore, the range of colors selected for remote sensing was adjusted for each quadrat (for example the RGB range for the quadrat 26 in March 2022 was: R: 0 to 0.45; G: 0 to 0.4; B: 0 to 0.5).

As yellow can easily be distinguished from the colour ranges of the rest of the plot, a conversion to a false-colour image is unnecessary. The following image workflow explains how the percentage of yellow was estimated:
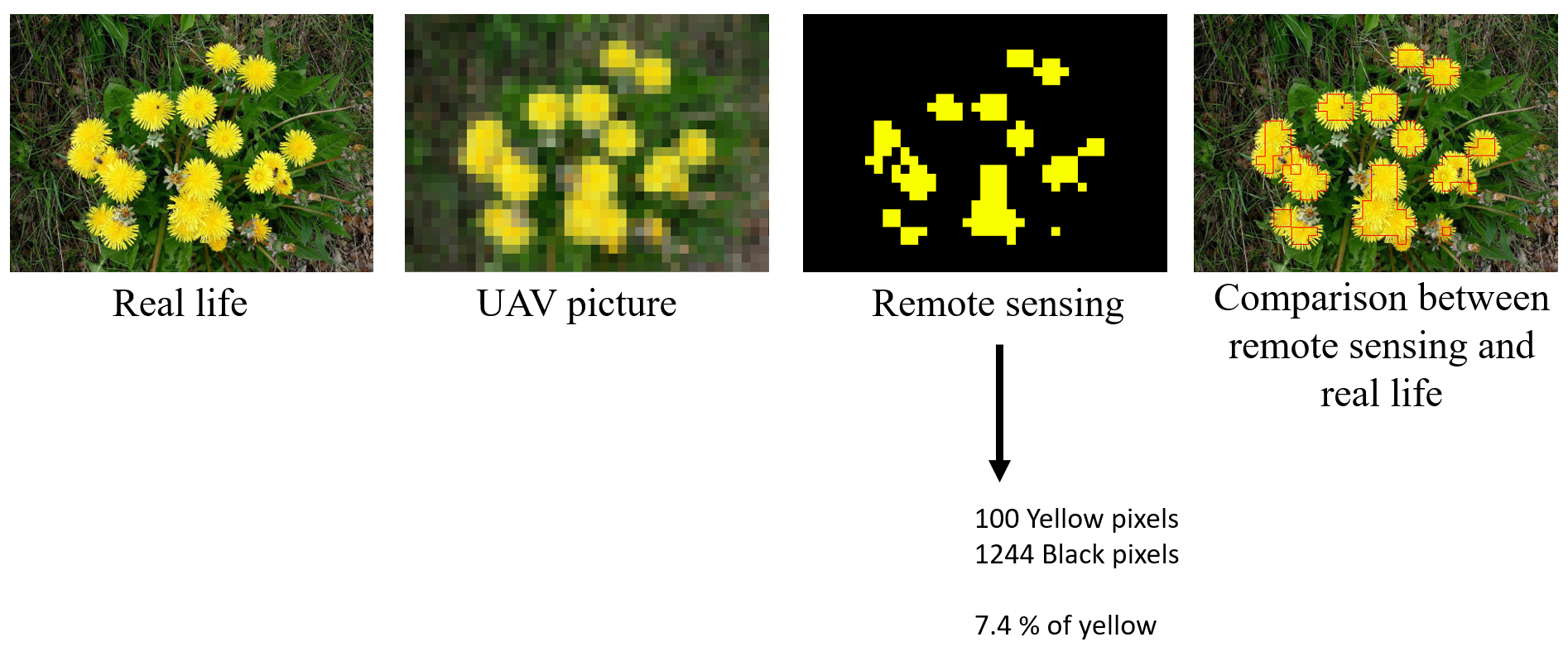


## **Reference**

*Hooper, S., Weller, H., & Amelon, S. (2020). Countcolors, an r package for quantification of the fluorescence emitted by pseudogymnoascus destructans lesions on the wing membranes of hibernating bats. Journal of Wildlife Diseases, 56.*

*Meyer, G. E., & Neto, J. C. (2008). Verification of color vegetation indices for automated crop imaging applications. Computers and Electronics in Agriculture, 63(2), 282‑293.*

**SuppInfo 4 - SUPPLEMENTORY RESULTS: Vole and dandelions densities index**

**
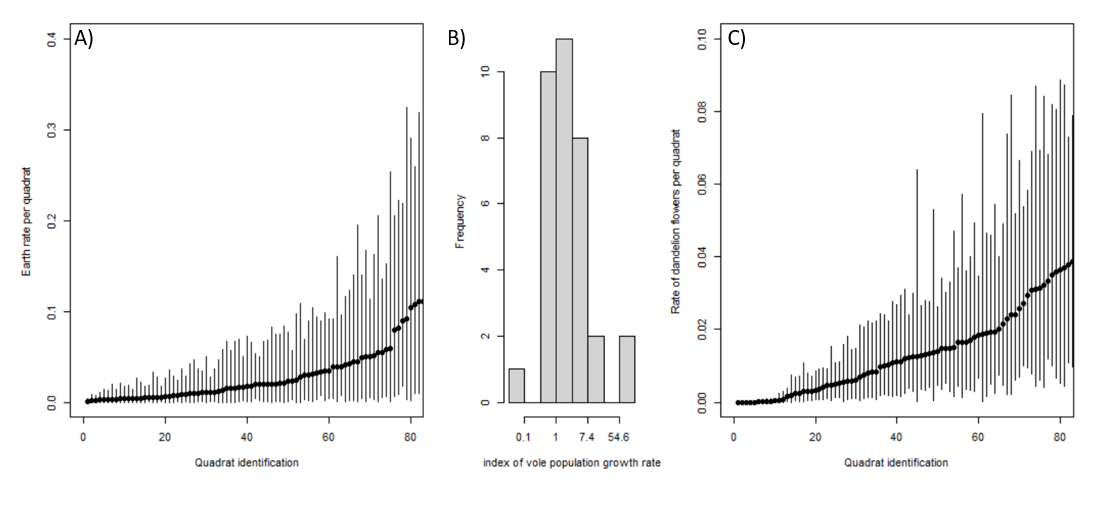
**

Figure 1: Variability of gross value dispersion A) Earth rate per quadrat in March 2021 and 2022 (median and interval with 90% of values), B) Vole population growth rate between March 2021 and March 2022, C) Dandelion density in May per quadrat (median and interval with 90% of values).

**SuppInfo 5 – SUPPLEMENTORY RESULTS : Distance sampling analyses between vole and dandelions**

**
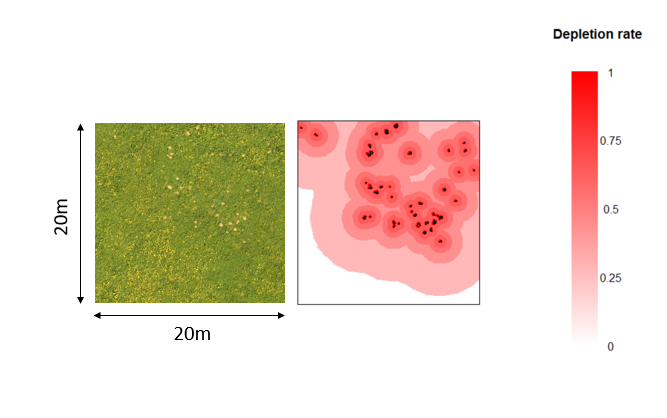
**

**b)**

**a)**

Figure 1: Example of depletion area of dandelions around vole mounds. a) drone image of the Quadrat GI0, extraction 1 in May 2022 (RGB), b) depletion rate within 0.5 m, 1 m, 2 m and 5 m buffers.
